# The Discrepancy of ANA and Compartment Bead Patterns Suggestive of a Neuropsychiatry Systemic Lupus Erythematosus (NPSLE)

**DOI:** 10.1155/2023/5260208

**Published:** 2023-10-26

**Authors:** Munawaroh Fitriah, Lita Diah Rahmawati, Indah Adhita Wulanda, Hani Susianti, Betty Agustina Tambunan

**Affiliations:** ^1^Department of Clinical Pathology, Faculty of Medicine, Universitas Airlangga—Dr. Soetomo General Academic Hospital, Surabaya, Indonesia; ^2^Department of Internal Medicine, Faculty of Medicine, Universitas University—Dr. Soetomo General Academic Hospital, Surabaya, Indonesia; ^3^Department of Clinical Pathology, Faculty of Medicine, Brawijaya University—Dr Saiful Anwar General Hospital, Malang, Indonesia

## Abstract

Neuropsychiatric systemic lupus erythematosus (NPSLE) exhibits neurological and psychiatric manifestations in systemic lupus erythematosus (SLE) patients, which NPSLE diagnosis can be challenging for rheumatologists. An Indonesian female, 44 years old, complained of two times seizures with 10-min duration, which during seizures were stiff, eyes rolled up, foaming at the mouth, wet the bed, and fainting afterward. The patient also has a history of SLE and received cyclophosphamide therapy 5 years ago. Her clinical condition showed facial and lingual palsy, with central type on the right. Antinuclear antibody indirect immunofluorescence (ANA IF) positive using cytobead ANA with a homogenous pattern and cytoplasmic speckled titer 1/80. Confirmation beads showed positive of dsDNA only. ANA profile showed positive antinucleosome, antihistone, and AMA-M2, and also increased anticardiolipin antibody that supports the diagnosis of NPSLE. The difference in the pattern of ANA IF with confirmation beads suggests the presence of other autoantibodies in NPSLE.

## 1. Introduction

Neuropsychiatric systemic lupus erythematosus (NPSLE) is a severe complication in systemic lupus erythematosus (SLE) patients characterized by neurological and psychiatric manifestations [1]. Primary NPSLE is increasingly prevalent in the population incidence rate of 0.5 per 100,000 persons/year [2]. NPSLE encompasses several neurological or psychiatric symptoms. When a possible diagnosis of NPSLE is considered, it must be supported by the primary diagnosis of SLE, and the possibility of non-NPSLE diseases, such as drug side effects and mental or functional conditions, must be ruled out. It is that 28%–40% of adult SLE patients' experince NPSLE before diagnosis and/or during SLE diagnosis. After 1 year of being SLE diagnosis, it was reported that 63% develop NPSLE [3].

Clinical manifestations of NPSLE range from localized or isolated to diffuse, peripheral nervous system (PNS), and/or central nervous system (CNS) [4]. According to the American College of Rheumatology, the manifestations of NPSLE can be divided into 12 CNS and seven PNS syndromes. Additionally, these were classified into focal neurological syndromes and diffuse neuropsychological syndrome. Manifestation of focal neurological syndromes of CNS is seizure disorder, aseptic meningitis, demyelinating syndromes, myelopathy, headache, cerebrovascular disease, and movement disorders. The diffuse neuropsychological syndromes of CNS are anxiety disorders, psychosis, acute confusional state, cognitive dysfunction, and mood disorders. The focal PNS manifestations are autonomic disorders, myasthenia gravis, polyneuropathy, cranial neuropathy, Guillain–Barre syndrome, mononeuropathy, and plexopathy [1].

Diagnosis of NPSLE can be challenging for rheumatologists due to the lack of specific and sensitive laboratory serum or CSF biomarkers, radiological imaging changes, and other formal criteria in establishing the diagnosis and guiding the treatment and management decisions in NPSLE [1, 5]. This case study showed the discrepancy between the antinuclear antibody (ANA) and compartment bead patterns in NPSLE diagnosis.

## 2. Case Presentation

A 39-year-old Indonesian woman complained of having had two seizures of 10-min duration. When the hand spasms are stiff, the eyes glance up, and the mouth is foaming and wetting the bed. After the seizure, the patient is unconscious. The patient had no history of previous seizures. The patient also has a history of SLE, diagnosed 5 years ago with high titer ANA and she received cyclophosphamide therapy. The patient had no experience of diabetes mellitus, hypertension, stroke, and tumor.

Her clinical condition showed facial and lingual palsy with central type on the right. Laboratory abnormalities included normochromic anemia (hemoglobin 10.6 g/dL, hematocrit 32.2%, MCV of 88 fL, MCH of 29 pg, MCHC of 32.9 g/dL) with slightly decreased platelet level (124,000 cell/*µ*L), slightly decrease of albumin level (3.4 g/dL), the elevation of erythrocyte sedimentation rate (72 mm/hr), and slightly decrease of potassium (3.4 nmol/L). Meanwhile, PT/APTT, AST, ALT, bilirubin, BUN, creatinine, glucose, iron, sodium, and chloride levels were normal. Urinalysis examination showed proteinuria (+2), hematuria (+1), an increased albumin/creatinine ratio of ≥300 mg/g, and a protein/creatinine ratio of ≥0.5 mg/g. Blood gas analysis showed metabolic acidosis (pH of 7.34, pCO_2_ of 31 mmHg, HCO_3_ of 16.7 mmol/L, and base excess of −9.1 mmol/L).

**Figure 1:**
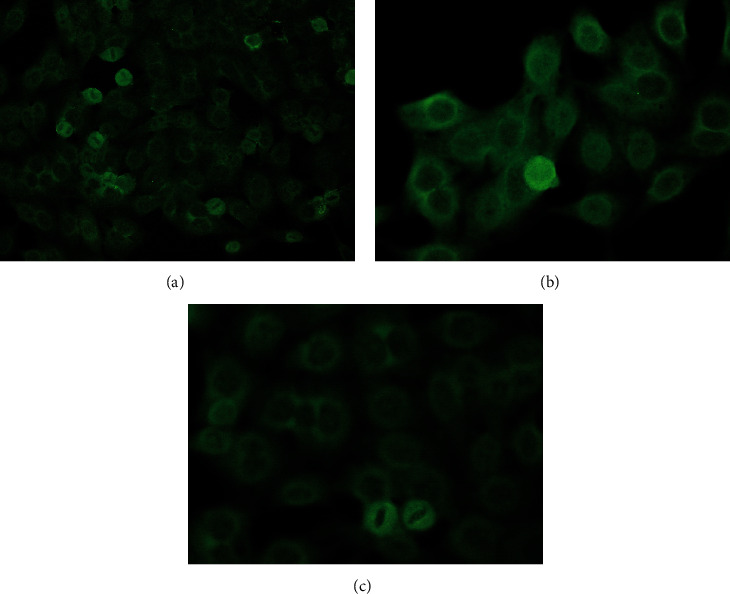
Homogeneous ANA IFA pattern—cytoplasmic speckled titer 1/80.

**Figure 2:**
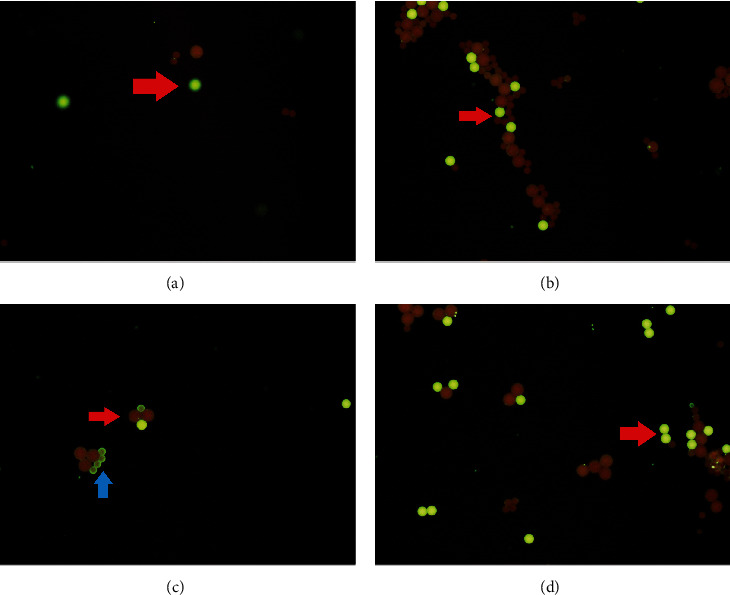
Results of ANA examination using the cytobead method. (a) Compartment 1 is negative (using tiny beads to detect anti-La/SSB and large beads for CENPB). (b) Compartment 2 is negative (using tiny beads to detect anti-Sm and large beads for RNP Sm). (c) Compartment 3 is positive for tiny beads, which means positive for anti-Ds-DNA (using small beads to detect anti-dsDNA and large beads for Scl-70). (d) Compartment 4 negatives (using tiny beads to detect anti-Ro-60 and large beads for RO-52). Red arrow: reference bead; blue arrow: positive beads.

Immunology results show an increase of procalcitonin (0.32 ng/mL), ferritin (427.6 mg/dL), positive ana test (ANA test ELISA index 392.66), low level of C3 (19 mg/dL) and C4 6 (mg/dL), elevated IgG anticardiolipin (91.10 GPLU/mL). Antinuclear antibody indirect immunofluorescence (ANA IF) positive using cytobead ANA with a homogenous pattern and cytoplasmic dense fine speckled titer 1/80 (Figure 1). In addition, the dsDNA test was positive (Figure 2). ANA profile was examined using euroline ANA profile three panels plus DFS70 showed strong positive for antinucleosome (++), antihistones (+++), anti-AMA-M2 (+++), and borderline for anti-RNP/Sm, PM-Scl100 (PM 100), and anti-dsDNA and negative for anti-Sm, SSA, Ro-52, SSB, Scl-70, Jo-1, centromere B, PCNA, and ribosomal protein (RIB).

A radiological examination showed several abnormal results. Electroencephalography indicates mild diffuse encephalopathy that may have general epileptogenic potential. Head multislice computed tomography with contrast showed expected no infarction, bleeding, mass, or infection in brain parenchyma. In addition, right ethmoidal sinusitis and minimal left maxillary sinusitis were found. Chest X-ray showed paracardial infiltrate, and heart showed no abnormalities.

This patient with epileptic seizures and abnormal encephalopathy findings on EEG was followed up in a hospital ward. She was diagnosed as NPSLE with lupus nephritis, anemia, and hypoalbuminemia complications. Consultation with the neurologist was performed during hospitalization, she received therapy myfortic of 2 × 360 mg, methylprednisolone of 1 × 8 mg, irbesartan of 1 × 50 mg, paracetamol of 3 × 500 mg, calk of 1 × 500 mg, folic acid of 1 × 1 mg, hydroxychloroquine of 1 × 200 mg, lansoprazole of 1 × 30 mg, and meloxicam of 15 mg (if needed). Patients often have flares within a few months after hospitalization, which indicates a poor prognosis. Her family does no have experience of seizure or SLE.

## 3. Discussion

The American College of Rheumatology identifies 19 neuropsychiatric syndromes in SLE patients that can be divided into CNS and PNS manifestations. CNS syndromes are more common than peripheral and can be further classified into diffuse or focal manifestations. Neurological manifestations are associated with decreased quality of life, high morbidity, and mortality rates. In particular, the lungs, kidneys, and CNS involvement can cause severe sequelae. Because there are no specific biomarkers of CNS activity, NPSLE diagnosis is often made by an exclusion diagnosis. The pathological mechanism in primary NPSLE is the direct effect of intrathecal inflammatory cytokines, disruption of the blood–brain barrier (BBB), and atherosclerosis and thrombotic vasculopathy due to antiphospholipid antibodies (APL). Neuronal dysfunction and apoptosis are caused by autoantibodies binding to neurons [6].

Various autoantibodies are associated with the pathogenesis of NPSLE. ANA results positively in 70%–90% of SLE and NPSLE patients [7]. High ANA titers are frequently observed among patients with NPSLE. Anti-ENA antibodies (a heterogeneous group) can target various antigens, including anti-Sm, anti-RNP, anti-SSA/Ro, and anti-SSB/La. NPSLE patients are positive for anti-ENA with an estimated 50%–60%. Whereas anti-Sm was detected in 18%–48% and anti-RNP in 18%–60% of all NPSLE patients. Anti-Sm antibodies are associated with BBB disruption and acute confessional state in NPSLE patients. An estimated 81% of NPSLE patients have anti-dsDNA detection, which anti-dsDNA is associated with poor visuospatial abilities, attention, and executive function [8]. Risk factors associated with NPSLE include CNS damage or SLE activity, previous neuropsychiatric events, and moderate to high titer of APL, such as lupus anticoagulant and anticardiolipin or anti-*β*2 glycoprotein 1 (IgG or IgM), particularly in cerebrovascular disease, seizures, myelopathy, and cognitive dysfunction. Meanwhile, antiribosomal P protein antibodies (anti-P antibodies) are associated with lupus psychosis [6].

Cytobead ANA IF combines the stages of IFA (cell-based assay) screening and confirmation of antibodies using a bead-based assay. The results of the antinuclear antibody immunofluorescence assay (ANA IFA) examination using cytobead, in this case, showed a mixed homogenous and cytoplasmic speckled pattern with a small bead in compartment 3 which was positive, indicating dsDNA [9]. Cytoplasmic dense fine speckled pattern can be positive in patients with SLE and antisynthetase syndromes, including interstitial lung disease, polyarthritis, and Raynaud's phenomenon. In patients suspected of having SLE with this pattern, it is necessary to proceed with the examination of the antiribosomal P phosphoprotein on the ENA profile. Anti-RibP is associated with neuropsychiatric lupus and autoimmune hemolytic anemia in SLE children. If there is clinical suspicion of antisynthetic syndrome, it is necessary to proceed with testing for anti-tRNA synthetase or anti-SRP [10, 11]. In this case, the ENA profile showed negative antiribosomal protein but strongly positive for antinucleosome, histone, and anti-AMA-M2.

Approximately 60% of NPSLE patients have autoantibodies to histones target the five major classes of histone proteins: H1, H2a, H2b, H3, and H4 [9]. They correlate to disease activity rather than neurological manifestation. However, several previous studies reported that specific autoantibodies, such as H1 and H3 fractions correlate with neuropsychiatric symptoms [12]. Using IIF on standard substrates of antihistone antibodies produces a homogeneous, chromosome-positive staining of the nucleus [13]. A significant association exists between simultaneous positivity to anti-DNA, antinucleosome, and antihistone antibodies and renal disease activities, especially in proliferative glomerulonephritis [14].

Antinucleosome antibodies (ANuA) react exclusively to nucleosomes, not individual histones or native nonprotein-complexed DNA [15]. In glomerulonephritis, nucleosomes bind autoantibodies to glomerular basement membranes with increased permeability and inflammatory response [13, 16]. It could be one of the most sensitive markers in the diagnosis of SLE, especially in anti-dsDNA-negative patients. Furthermore, there is a significant correlation between the level of antinucleosome antibodies and lupus disease severity. ANuAs are better for predicting flares in quiescent lupus [17].

APL target anionic phospholipids and protein phospholipid complexes. Specifically, APL targets the negatively charged phosphatidyl glycerol, phosphatidyl serine, phosphatidyl acid, inositol phosphatides, and cardiolipin, in addition to the neutrally charged phosphatidyl ethanolamine, phosphatidyl choline, platelet-activating factor, and sphingomyelin. Studies have shown that neuronal damage can occur directly APL-mediated without ischemia. Persistent anticardiolipin antibodies are significantly associated with cognitive impairment [18].

Epilepsy occurs in 12%–22% of SLE patients and is significantly associated with morbidity and mortality. Single-isolated and tonic–clonic seizures are the most common (67%–88%) types. EEG abnormalities are often found in SLE (60%–70%). The epileptiform EEG pattern shows a tendency for seizures to recur. Inflammatory processes play a significant role in the epileptic seizures pathogenesis. Meanwhile, ischemic vascular disease and cerebral tissue-binding antibodies (anticardiolipin and anti-Sm) have been associated with seizures. Epilepsy in SLE has also been associated with APL, disease activity, and some manifestations of NPSLE (psychosis, stroke) [6]. This study is in line with the report of Appenzeller et al. [19] involving 60 patients with epileptic seizures. Epileptic seizures occurred at the onset of SLE symptoms in 19 (31.6%) and after the onset of SLE in 41 of 60 (68.3%) patients. Fifty three of 60 (88.3%) patients had acute symptomatic epileptic seizures, and seven of 60 (11.7%) had recurrent epileptic seizures. The variables associated with acute epileptic seizures at the onset of SLE were stroke (*p* < 0.001) and APL antibodies (*p*=0.001). Epileptic seizures during monitoring were found to be associated with nephritis (*p*=0.001), APL antibodies (*p*=0.005), and epileptic seizures at disease onset (*p* < 0.001). The seven patients with recurrent epileptic seizures had APL syndrome and epileptic interictal abnormalities on EEG [19]. The pathological mechanisms in NPSLE are the direct effect of intrathecal inflammatory cytokines, disruption of the BBB, atherosclerosis, and thrombotic vasculopathy due to APL. Autoantibodies bind to neurons, causing neuronal dysfunction and apoptosis [6].

The patient was diagnosed with SLE 5 years ago, with a high ANA ELISA titer. From the ANA IFA examination, a mixed homogeneous pattern and cytoplasmic speckled with dsDNA, antihistone, antinucleosome, AMA-M2, RNP Sm, and PM-Scl 100 were positive and also high positive for anticardiolipin antibody, low C3 and C4 levels. According to the previous literature, this finding suggested increased of disease activity, and possible epileptic seizure as a manifestation of NPSLE associated with lupus nephritis [20]. Examination of specific autoantibodies to the brain component cannot be carried out and is a limitation in this case study.

## 4. Conclusion

NPSLE is established based on diagnosing SLE with neuropsychiatric symptoms and excluding other non-NPSLE causes. An increased of cardiolipin antibody and mixed homogenous nuclear and cytoplasmic speckled ANA pattern with bead confirmation in the positive anti-dsDNA compartment, positive antinucleosome, antihistone, and AMA-M2 from ANA profile result supports the diagnosis of NPSLE. ANA IFA patterns with disproportionate confirmatory beads require further confirmatory testing to look for other autoantibodies.

## Data Availability

The datasets used and/or analyzed during the current study are available from the corresponding author on reasonable request.
